# Acute Phase Protein Levels as An Auxiliary Tool in Diagnosing Viral Diseases in Ruminants—A Review

**DOI:** 10.3390/v10090502

**Published:** 2018-09-15

**Authors:** Daria Reczyńska, Magdalena Zalewska, Michał Czopowicz, Jarosław Kaba, Lech Zwierzchowski, Emilia Bagnicka

**Affiliations:** 1 Institute of Genetics and Animal Breeding, Polish Academy of Sciences, Postepu 36A str., 05-552 Jastrzębiec, Poland; d.reczynska@ighz.pl (D.R.); m.zalewska@ighz.pl (M.Z.); l.zwierzchowski@ighz.pl (L.Z.); 2 Laboratory of Veterinary Epidemiology and Economics, Faculty of Veterinary Medicine, Warsaw University of Life Sciences, Nowoursynowska str. 159c., 02-776 Warsaw, Poland; Michal_czopowicz@sggw.pl (M.C.); jaroslaw_kaba@sggw.pl (J.K.)

**Keywords:** dairy ruminant, acute phase protein, viral infection

## Abstract

We examined acute phase protein (APP) concentrations in viral infections of dairy ruminants and assessed the potential role of characteristic patterns of APP changes in auxiliary diagnosing viral diseases. All viruses reviewed are common causes of farm animal diseases. APPs are among the first agents of immunity, and their concentrations could be diagnostically relevant. In the most common ruminant viral diseases, elevated serum amyloid A (SAA) and haptoglobin (Hp) levels in blood serum have been observed. However, since these proteins are the main APPs in many viral infections, it is impossible to use their levels for diagnosing particular infections. Decreased Cp and albumin expression could help differentiate the bluetongue virus infection from other diseases. Lastly, analysis of SAA levels in blood serum and milk could be helpful in diagnosing small ruminant lentivirus infection. While promising, APP levels can only be considered as an auxiliary tool in diagnosing viral diseases in ruminants.

## 1. Introduction

In developed countries, the welfare of animals has become a very important issue for conscientious consumers. In addition to animal rights concerns, the health of the animal affects the quality of the product. For many years, domestic animals were selected only for their improved production traits. Functional traits, including resistance to infections, have only recently been considered in breeding programs (except in Scandinavian countries) [1]. As a result, high-yield animals are often prone to infections. Viruses are a common cause of farm animal infectious diseases, which generate considerable economic losses worldwide. Thus, prevention and early diagnosis of viral infections are vital. The acute phase response (APR) is an organism’s first line of defense against infections, injury, or trauma, with acute phase proteins (APPs) as its agents. APPs comprise a large, heterogeneous group of proteins and polypeptides, whose concentration changes during many diseases, including during viral infections. One potential way of diagnosing a disease/virus in a farm animal is by determining the concentration of APPs [2]. Knowledge about how APPs, as mechanisms of immune defense, are produced in response to viral infections in farm animals may be helpful in the early diagnosis of viral infections.

## 2. Acute Phase Proteins

The immune system, activated by contact with pathogens, allows an organism to defend itself against infections. Physical and chemical barriers, activation of the complement system, phagocytic and natural killer (NK) cells, and the production of cytokines are the first line of immune defense, termed innate immunity [3]. APPs belong to a heterogeneous group of proteins and polypeptides that constitute the first line of defense and are related to APR, which occurs shortly after pathogenic agent invasion. The concentration of APPs in serum may indicate acute or chronic infection. However, APR also occurs in other conditions unrelated to pathogens, such as in neoplastic diseases, trauma, or various inflammatory reactions [4]. APPs are responsible for triggering and then amplifying or weakening defense mechanisms, thus they have both pro- and anti-inflammatory properties. Furthermore, they may differ significantly in terms of their role in the organism, mode of action, and structure. Their production occurs mainly after the complement system has been activated. APPs are also activated by pro-inflammatory cytokines, such as interleukin 1 (IL-1), interleukin 1 alpha (IL-1α), interleukin 1 beta (IL-1β), interleukin 6 (IL-6), and tumor necrosis factor alpha (TNF-α) [5].

In farm animals, several proteins have been recognized as APPs, which can be divided into two groups: (1) Positive APPs, whose concentrations increase during inflammation—serum amyloid A (SAA), haptoglobin (Hp), ceruloplasmin (Cp), fibrinogen (Fb), C-reactive protein (CRP), alpha-1-acid glycoprotein (AGP), alpha-1 antitrypsin (AAT), hemopexin (Hpx), and lipopolysaccharide binding protein (LBP); and (2) negative APPs, whose concentrations decrease during inflammation—albumins, transferrin (Tf), and transthyretin (TTR) [6,7,8]. Positive APPs are further divided into three subgroups based on the increase in their concentration and the time period after infection: (1) Major—the concentration increases 10- to 100-fold in the first 48 h after infection; (2) moderate—the concentration increases 2- to 10-fold in the first 3–4 days post infection; and (3) minor—the concentration increases only slightly and change occurs more slowly [9]. Depending on the animal species, the various aforementioned proteins are considered APPs.

Serum amyloid A (SAA) belongs to the group of apolipoproteins whose main roles are the binding, transporting, and scavenging of lipoproteins. Moreover, this protein is also a part of the immune response via neutrophil and macrophage activation or killing of coliform bacteria. Other immune-related functions include stimulating the migration and adhesion of T cells, participating in monocyte chemotaxis, inhibiting phagocyte oxidative burst, aggregating platelets, and driving lymphocyte and endothelial cell proliferation. Moreover, SAA is responsible for detoxifying endotoxins and regulating phagocytosis during inflammation and infection. In farm animals, an increased concentration of SAA has been confirmed during trauma, viral infections, and physical stress. During mastitis, an elevated level of SAA has also been observed [10,11,12]. SAA belongs to the group of major APPs in cattle and small ruminants [9].

Haptoglobin (Hp) is a positive APP produced mainly by hepatocytes, but has also been identified in skin, lung, and kidney cells. The main role of Hp is the binding of free hemoglobin (Hb), thus forming Hp-Hb complexes that are non-toxic to living cells (in contrast to free Hb, which has oxidative properties). The binding of free Hb by Hp also prevents iron-utilizing bacteria from using Hb-derived iron (Fe) (from hemolysis). The Hp-Hb complex is then eliminated by the reticuloendothelial system [13]. An increased level of free Hb, after stimulation by pro-inflammatory interleukin 6 (IL-6), induces the formation of Hp-Hb complexes, which are subsequently internalized by CD163 receptors on the cell surface of monocytes and macrophages. As a consequence, it induces secretion of the anti-inflammatory interleukin-10 (IL-10). Moreover, after the delivery of Hp-Hb complexes to macrophages, the heme molecule of hemoglobin is degraded by heme oxygenase 1 into the anti-inflammatory metabolites (CO, biliverdin, and Fe^2+^). Moreover, the Hp-Hb complex regulates the bioavailability of nitric oxide (as a nitric oxide scavenger), which is considered a blood flow regulator. In addition, Hp can act as a vascular homeostasis and angiogenesis regulator [14,15]. Free Hb has strong oxidative activity, thus it is important that it binds into harmless complexes to prevent tissue damage caused by oxidative stress [16]. Hp is a major APP in cattle and small ruminants [9].

Ceruloplasmin (Cp) is a positive APP involved in metal homeostasis. This protein is synthesized mainly in the liver, but also in other organs, such as the spleen, lung, testis, and brain. It is difficult to determine the major function of this protein in particular physiological conditions. Cp binds up to 95% of copper ions in blood serum—it can tightly bind six coppers, and it can also bind up to an additional ten atoms, but less tightly. However, changes in copper concentrations do not affect Cp production. Another important role of Cp is ferroxidation, or facilitating the oxidation of Fe^2+^ to Fe^3+^, a step that is required for transferrin to further use Fe^3+^. Cp is also responsible for reducing oxygen substrates without releasing free oxide radicals and H_2_O_2_ as mediates. Moreover, Cp shows oxidative activity toward substituted organic compounds. It has been shown to work as an amine oxidase. As suggested by the aforementioned examples, Cp is considered a major contributor to plasma antioxidant capacity [17]. Cp is a minor APP in small ruminants [8,18].

The next protein involved in organism homeostasis maintenance is fibrinogen (Fb), a fibrin precursor. It is built from three chains (alpha, beta, and gamma) coded by three separate genes. This protein belongs to a group of soluble glycoproteins that are synthesized mainly in the liver and is responsible for blood clotting and tissue repair [6,19]. Thus, after blood vessel wall disruption, Fb functions as a coagulation protein involved in the coagulation cascade, which leads to clot formation and then to soft tissue repair [20]. Moreover, it binds to the CD11/CD18 receptors on phagocytes and subsequently starts a cascade of signals that leads to an immune response (increased degranulation, phagocytosis, and antibody-dependent cellular cytotoxicity, and delayed apoptosis). Fb can also regulate blood viscosity and blood flow. It is a moderate APP in cattle and goat and a minor APP in sheep [8,9].

The first described APP was C-reactive protein (CRP), a positive APP identified in humans [21]. CRP in general binds to pathogens, enhancing their opsonization, before the specific immunoglobulin (IgM and IgG) are produced. This protein also interacts with necrotic or apoptotic cells and nuclear material. CRP belongs to the pentraxin family of proteins (built from five identical, non-glycosylated subunits consisting of 206 amino acid residues each and organized in a symmetric disc-shape structure), which are highly evolutionarily conserved from arachnids to mammals. Each subunit consists of two parts: (1) The recognition site, which is responsible for ligand binding (a phosphocholine (PCh) binding site consisting of two calcium ions in the hydrophobic pocket); and (2) the effecting site, which, after binding with a particular ligand, interacts with the complement system (C1q) and initiates the classical complement pathway and immunoglobulin IgG receptors (Fcγ) [22,23]. CRP is mainly produced by hepatocytes, but can also be produced by neurons, atherosclerotic plaques, monocytes (including peripheral blood monocytes), Kupffer cells, macrophages, lymphocytes, smooth muscle cells, kidney cells, and alveolar cells. Moreover, CRP degradation results in the formation of several bioactive peptides that inhibit the pro-inflammatory and tissue-destructive activity of neutrophils. CRP also impairs adhesion of neutrophils to the endothelium, which reduces transport into this tissue and leads to inhibition of the pro-inflammatory response [24]. CRP is a moderate APP in cattle and sheep [9]. CRP activity in goats has not been fully elucidated; there is one report showing changes in the concentration of CRP in this species, but only during pneumonia [25].

Another positive APP is alpha-1-acid glycoprotein (AGP), which is synthesized mainly by hepatocytes, though extrahepatic secretion of this protein has been also confirmed (by epithelial and endothelial cells). From a structural point of view, AGP belongs to lipocalin, a group of proteins binding small hydrophobic molecules, e.g., heparin, histamine, serotonin, steroids, catecholamines, or pharmaceuticals. AGP is responsible for transporting substances in plasma and modulating the immune response, but it also works as a chaperone, protecting organisms against bacteria. AGP assists in overcoming bacterial infections as a non-specific antibacterial agent by neutralizing the toxicity of lipopolysaccharide (LPS), a component of the cell envelope of Gram-negative bacteria. AGP also acts as an anti-inflammatory agent, inhibiting neutrophil activation [26,27]. Moreover, it inhibits lymphocyte proliferation induced by mitogen as well as activity of NK cells [10]. This protein reduces tissue damage caused by excessive complement system activity, reduces the apoptosis rate in some tissue, and is responsible for extending the monocyte lifespan [28]. Furthermore, AGP inhibits phagocytosis and B- and T- cell maturation, and downregulates chemotaxis in monocytes. It is also necessary for maintaining local homeostasis and increases the endothelial barrier permeability of capillaries [29]. AGP belongs to the group of moderate APPs in cattle, goats, and sheep [8,9].

Alpha-1 antitrypsin (AAT) is a serine protease inhibitor, which belongs to the serpine protein family. Its main biological role is protecting tissues at the site of inflammation from proteolytic enzymes produced by neutrophils. Moreover, it inhibits the activity of NK cells [6,8,10]. AAT belongs to the group of minor APPs in cattle [30].

The glycoprotein hemopexin (Hpx or Hx), also known as beta-1B-glycoprotein, is also a positive APP. Together with Hp and Tf, it participates in iron homeostasis. Its main function is binding free heme after red blood cells have been disrupted and therefore it plays an antioxidant role. Moreover, Hpx binds nitric oxide and carbon monoxide to neutralize its toxic potential. It is also suggested that Hpx may exert an immunoregulatory effect by activating different signaling pathways [31]. Currently, it is known only to play a role in protozoan infection (*Trypanosomavivax*) in sheep [8].

Lipopolysaccharide binding protein (LBP) is a glycoprotein produced mainly by hepatocytes, but it is also expressed extrahepatically in the gastrointestinal tract, lung, female reproductive system, thyroid gland, nervous system, and mammary gland of cattle. It defends against bacterial infections; however, its role in viral infections is unknown. Its main role is to bind bacterial LPS and to present this compound to the receptors of antigen-presenting cells (e.g., CD14, CD11/18), which are present on the membrane of immune system cells, such as monocytes, macrophages, and granulocytes. LBP not only binds to LPS, but also to lipoteichoic acid, a component of the wall of Gram-positive bacteria, such as *Streptococcus pneumonia* and *Staphylococcus aureus*. Whether LBP exerts a positive or negative effect on the immune response depends on its concentration. It is pro-inflammatory at low concentrations, for example, in activating mononuclear cells. Conversely, it is anti-inflammatory at high concentrations, for example, in inhibiting LPS-induced cellular activity. Interestingly, LBP can also act as an opsonin against *Salmonella* spp. and *Klebsiella pneumoniae,* facilitating their phagocytosis. [7,8,30]. LBP belongs to the moderate group of APPs in cattle during *Mannheimia haemolytica* infection [30]. Its role in sheep and goats remains unclear.

Negative APPs are those whose concentrations decrease during inflammatory processes. The biological function of negative APPs is the transport of organic and inorganic substances.

The main roles of albumins, which are negative APPs, are to maintain plasma osmotic pressure and, thus they have many binding sites to transport biologically active compounds both natural and synthetic ligands, e.g., free fatty acids, calcium, magnesium, bilirubin, thyroxin, hormones, and pharmaceuticals. Albumins also serve as a reservoir of amino acids for positive APP synthesis. If a pathological state occurs, amino acids are redirected to positive APP production instead of albumin synthesis, thus downregulating albumin production [6,30,32]. Albumins are produced mainly in the liver, but lactalbumin (LALBA) is secreted by mammary gland secretory epithelial cells. LALBA’s main function is catalyzing the synthesis of lactose. However, its anti-tumor activity (via induction of tumor cell apoptosis) and bactericidal activity have also been reported [32].

One negative APP is transferrin (Tf), produced by the liver, whose main function is transporting iron ions, mostly to growing cells. Substantially, all circulating in plasma iron is reversible bonded to transferrin. Moreover, in normal body conditions, Tf prevents toxicity of iron-mediated free radicals. During bacterial infections, Tf limits iron-utilizing pathogens from accessing this metal, which is crucial for their growth. A negative link between hemoglobin and Tf has been reported, and the concentration of Tf is higher in young animals compared to their adult counterparts [6,7].

Another negative APP is transthyretin (TTR), also known as thyroxin-binding protein. Its main role is participating in the thyroid hormone transport pathway and creating a complex with retinol-binding protein. These two proteins are associated indirectly with vitamin A transport in plasma. Moreover, TTR binds retinol. TTR is a precursor protein often found in amyloid deposits and is associated with many amyloid diseases [6]. TTR concentrations change in response to heat stress and bacterial and viral infections [33]. So far, Tf and TTR are not considered as APP in small ruminants.

Monitoring APP levels can contribute insights into the etiology of infectious diseases and can facilitate finding the best models for conducting research on those diseases [9]. Moreover, APPs are considered indicators that, in many situations, can assist in identifying an organism’s infection type (e.g., bacterial, viral, or parasitic) [4].

## 3. APP Levels in Blood Serum of Farm Dairy Ruminants During Viral Infections

Since an animal’s welfare affects its productivity, factors that impact welfare have recently become the subject of intense study. One factor that negatively affects animal welfare is infection, be it viral, bacterial, parasitic, or fungal. Understanding how an organism fights pathogen is essential for the proper prevention and treatment of infection. APPs are one of the agents in the first line of immune defense, thus it is crucial to fully understand their activity patterns. The current state of knowledge on APPs as an indicator of viral infection in ruminants is presented in Table 1.

Bovine respiratory disease (BRD), which affects mostly calves, is one of the most life-threatening diseases for cattle and is responsible for huge economic losses for the beef and dairy industries. Together with *Mannheimia haemolytica* (Gram-negative bacteria), *Pasteurella multocida* (Gram-negative bacteria)*, Haemophilus somnus* (Gram-negative bacteria), and *Mycoplasma bovis* (Gram-positive bacteria)*,* a bovine respiratory syncytial virus (BRSV) is the cause of this disease. This virus belongs to the *Pneumovirus* genus, *Paramyxoviridae* family. It has single negative-strand RNA, and its replication occurs in epithelial cells and type II pneumocytes. Viral transmission occurs by direct contact with infected animals or indirectly via contaminated surfaces. The symptoms of this disease are high fever, cough, rhinitis, decreased appetite, anorexia, and problems with respiration (wheezing) [47,48,49]. Heegaard et al. (2000) [39] reported that infection of calves with BRSV led to changes in SAA and Hp concentration levels, and their levels were linked to the severity of the disease. The maximum concentrations of Hp and SAA in serum were observed in the first week post-infection. The maximum concentration of SAA in the sera of infected animals was 5- to 7-fold higher than the SAA concentration in the non-infected animals’ sera. Changes in Hp concentration were analogous to those of SAA.

Bovine viral diarrhea virus (BVDV) belongs to the *Pestivirus* genus, *Flaviviridae* family. Viruses from this group contain single-stranded RNA [50] and induce disorders of the reproductive system, such as infertility, abortion, stillbirth, fetal malformation, and fetal mummification, in cattle. During infection with BVDV, animals gain weight and their milk production decreases. Moreover, BVDV causes diarrhea and immunosuppression. Transmission of BVDV occurs via contact with infected animals and through the placenta, from infected mother to offspring [51,52,53]. A few researchers have shown that when calves are infected with BVDV, their concentrations of SAA, Hp, and Fb in blood serum changed [40,41]. The Hp concentration was highest eight days post-infection (dpi) (with an approximately 2.5-fold increase), while the SAA concentration reached its peak 12 dpi (with a 5- to 6-fold increase) [42]. Gånheim et al. (2003) [43] obtained similar results: the SAA’s maximum concentration increased 4- to 5-fold at 8–9 dpi; Hp doubled its maximum concentration at 9 dpi; and Fb reached its maximum concentration at 8–9 dpi.

Foot and mouth disease virus (FMDV) belongs to the Aphthovirus genus, *Picornaviridae* family. It causes foot and mouth disease (FMD) in both wild and farm ruminants, such as cattle, goats, and sheep, as well as in pigs. The single-stranded RNA is located in a virus capsid. Prevention of FMD is crucial because the disease negatively affects animal productivity and compromises the quality of animal products [54]. The clinical symptoms of infection are high fever, loss of appetite, anorexia, depression, and dyspnea, lesions on the oral mucosa, intense salivation and lameness. The transmission of FMDV occurs very easily via several routes, e.g., via wind, barn environment, and contact with infected animals. Because FMD spreads very quickly in the herd and in the surrounding area, the eradication of the disease is extremely difficult. “Carrier phenomenon” also occurs with this disease: After a short acute phase of FMD, animals can still harbor the (symptomless and undetectable) infection and can continue to spread the virus [55]. Replication of this virus occurs mostly in epithelial cells, pharyngeal mucosa, and sometimes in lung cells. In blood serum of infected cows, increased levels of Hp, SAA, Fb, and Cp can be observed. The mean SAA concentration in infected animals is approximately eight times higher than that in uninfected animals. Moreover, the Hp concentration increases 5-fold during infection, while the concentrations of Cp and Fb increase approximately 2-fold [44,45]. Merhan et al. (2017) [46] also observed increased concentrations of SAA, Hp, and Cp in infected animals (approximately 6- to 9-fold, 3- to 4-fold, and 2-fold, respectively, depending on the severity of the disease), as well as decreased concentrations of albumins.

One of the most common goat and sheep pathogens is small ruminant lentivirus (SRLV), belonging to the *Retroviridae* family. This single-stranded RNA virus infects monocytes, macrophages, and epithelial and dendritic cells. The infection is lifelong, and it can persist in a latent form for months or years [56,57,58]. This multisystemic inflammatory disease causes arthritis of the carpal joints and (rarely) of the tarsal joints in adult goats, and respiratory distress, mammary induration, and occasionally neurological symptoms (progressive demyelinating encephalomyelitis) in sheep. Moreover, in both species, non-suppurative and indurative mastitis may occur. In young goats (i.e., kids), leukoencephalomyelitis can occur, but only in extremely rare cases [59,60,61]. Clinical signs of SRLV infection negatively impact animal welfare [62]. SRLV is transmitted vertically (via intake of colostrum or milk from the infected mother) and horizontally (via direct contact with body secretions of infected animals) [63]. SRLV infection causes economic loss due to the premature culling of infected animals and decreased milk yield [57]. Kaba et al. (2011) [64] showed no difference in the level of Hp between infected and uninfected goats; however, they did not distinguish between infected animals with the clinical and subclinical forms of caprine arthritis encephalitis (CAE). Conflicting results were reported by Czopowicz et al. (2017) [34], as in this study, they divided animals into three groups: SRLV-free, CAE-asymptomatic, and CAE-symptomatic. They noticed higher levels of SAA (a 5-fold increase) and Hp (a 2-fold increase) in the blood serum of CAE-symptomatic goats compared to CAE-asymptomatic and SRLV-free goats. Furthermore, they did not report differences in SAA and Hp levels in blood serum between goats free from infection and from those infected with SRLV without clinical symptoms of CAE. However, they noted a substantially lower level of these proteins in healthy goats than in those with clinical symptoms. They concluded that this difference was not due to the presence of the virus, but to inflammation. However, Reczyńska et al. [65] did find an elevated level of SAA in the blood serum of SRLV-infected goats without clinical signs of CAE, as well as a simultaneous decrease of SAA and Cp levels in the milk of those animals. These results suggest that an analysis should be done in both types of biological material in parallel to diagnose CAE in animals without clinical symptoms of the disease.

Border disease (BD) is an endemic viral disease in goats, sheep, and wild ruminants. The infection is induced by a single-stranded RNA virus named border disease virus (BDV). It belongs to the *Pestivirus* genus, *Flaviviridae* family. This infection has been reported in small ruminants in Japan [66], Austria [67,68], Turkey [69], the Netherlands [70], and China [71,72]. BDV has also been found in goats from Italy [73], and sheep from India [74] and Spain [75]. Infection can be transmitted horizontally and vertically. In small ruminants, the consequences of BDV infections are infertility, abortion, stillbirth, neonatal mortality, and offspring malformations, body tremors, and locomotor dysfunction [68,69,76]. Moreover, in infected animals, multisystemic clinical symptoms, such as hairy fleece, slow growth, digestive system dysfunction (diarrhea, hemorrhage in the stomach and in the small and large intestine], and reproductive system dysfunction (abortion and malformation] have been observed [71,72]. During infection, the quality of the sheep and goat products, such as meat, wool, and milk, decreases significantly, which can cause serious economic losses [76]. Balikci et al. (2013) [35] reported that average SAA and Hp concentrations were higher in the blood serum of goats infected with BDV compared to that of non-infected goats. The authors divided the tested animals into groups depending on the occurrence of abortion. For the non-aborting goats, SAA and Hp levels increased approximately 7- to 8-fold in infected animals compared to infection-free ones, while for aborting goats, SAA and Hp levels in infected animals were 11-and 15-fold higher, respectively, than those of uninfected animals.

Peste des petits ruminants virus (PPRV) belongs to the *Morbillivirus* genus, *Paramyxoviridae* family. It is an endemic viral infection in goats that can also occur in sheep, buffaloes, and other wild ruminants. This single-stranded RNA virus causes neutrophil dysfunction. The first clinical symptoms of infection are high fever and lack of appetite. Moreover, in infected animals, pneumonia, diarrhea, and rhinitis can be observed. Prevention of this disease is essential because of the high mortality rate among infected animals (as an acute phase reaction can lead to death) [77,78,79]. The virus is transmitted by close contact with infected individuals. Arslan et al. (2007) [36] reported that in blood serum of PPRV-infected sheep, the average concentrations of SAA and Hp increased two-fold compared to infection-free animals.

Bluetongue virus (BTV) has double-strand DNA and induces bluetongue disease (BT) in farm and wild ruminants. It belongs to the *Orbivirus* genus, *Reoviridae* family [37]. BTV affects lymphocytes, macrophages, and dendritic and endothelial cells, i.e., cells that generate innate and adaptive immune responses. In the late stage of BTV infection, viruses avoid neutralization by the immune system via intimate association with the membranes of circulating blood cells. This phenomenon allows for an extended duration of BTV infection. Transmission of the virus occurs via certain species of blood-sucking insects from the genus *Culicoides*. The characteristic symptoms of BT are high fever, nasal discharge, hemorrhage in the pulmonary and digestive tracts, ulcers in the oral cavity, edema, and effusion in the lungs and abdominal wall [80,81]. Sánchez-Cordónet et al. (2013) [38] reported increased levels of Hp (16- to 18-fold at 10–12 dpi) and SAA (3.5-fold at 2–15 dpi) in the BTV-infected sheep compared to infection-free sheep. Aytekin et al. (2015) [37] reported no differences in the albumin levels in blood serum of BTV-infected sheep compared to that of infection-free animals. These authors also showed that the Cp level was slightly lower (approximately 1.5-fold) in the BTV-infected group compared to the infection-free group.

## 4. Discussion

Hp and SAA are considered the major APPs for ruminants [8]. However, they have mostly been analyzed with respect to bacterial infections or multisystemic diseases. In this review, we focused on APP concentrations during different viral infections of livestock animals. In almost all the viral infections described above (BDV, PPRV, BTV, BRSV, BVDV, and FMDV), increased production of SAA was observed. The main role of SAA in organisms is lipid binding, and it has been suggested that lipids play a crucial role in the defense against viral infection. During viral infection, high-density lipoprotein (HDL) is transported directly to sites of damaged cells and tissues to repair them [82]. The response of other APPs, such as Hp or Cp, depends on the virus type. Both Hp and Cp participate in iron homeostasis (i.e., they have binding sites for iron ions). One hypothesis for what causes this phenomenon is that the free iron ions that accumulate during viral infection are cytotoxic to immune cells. High levels of free iron can cause neoplasia, overload of macrophages, and lipid peroxidation (damage to the cell membrane) [83]. An increased level of Hp in SRLV, BRSV, BVDV, BDV, PPRV, FMDV, and BTV could be evidence of the organism’s defense against the toxic activity of free iron ions. Therefore, active immune reactions are necessary to remove the damaged cells by phagocytosis [84]. One of the main roles of Cp is participation in copper metabolism. Copper can activate the production of reactive hydroxyl radicals, which can interfere further with different cellular events (e.g., lipid peroxidation, DNA damage, and apoptosis). Some researchers have shown that via this mechanism, Cp can even inhibit viral replication [85]. Since Hp and Cp are tissue damage markers, their concentrations should be higher in diseases that cause extensive tissue impairment (e.g., BVDV and FMDV [86]. During BTV and FMDV infections, the level of the albumin LALBA (a negative APP) was decreased). Aggregation of LALBA can cause caspase activation, which participates in cell apoptosis and immune response, e.g., the post-translational modification of cytokines [32]. Moreover, APP concentrations change during bacterial and parasitic infections. Infection of sheep with *P. multocida*, the Gram-negative bacterium, increased the concentration of Hp (34-fold), SAA (7-fold), and Fb (2-fold) [87], while infection of sheep with *Sarcoptes scabei* (an arthropod, the itch mite) caused the elevation of the concentration of Hp (2-5-fold), AGP (10-fold), and Cp (2-5-fold). Serum SAA, Hp, LBP, and AGP concentrations increased (approximately 10-150-fold; 10-30-fold; 2-6-fold; 2-10-fold, respectively) in goats infected with *Haemonchus contortus* (an abomasal blood-sucking nematode, the barber’s pole worm, one of the most pathogenic and common parasites of ruminants) [88]. Infection with Gram-negative or Gram-positive bacteria elevated SAA concentration in the mammary epithelial cells of dairy cattle: 18-fold and 12-fold, respectively, while was Hp approximately 1.2-fold. Moreover, an increase of CRP, LBP, and Lf concentration (approximately 10-, 15-, and 30-fold, respectively) was observed during mastitis in cows [89]. These results show that APP reach different levels in different infections. Therefore, it is important to know not only which proteins have changed, but also what concentrations they have reached. The information on the APPs concentrations in serum of healthy small ruminants was gathered by Iliev and Georgieva (2018) [8]; this paper presents APP concentrations in sheep, goat, and cattle, both healthy and infected with various viruses (Table 2).

## 5. Conclusions

There is a need to find markers so that infections can be quickly identified, in their subclinical state, and infected animals can be isolated from others or even eliminated from production to prevent the spread of the disease. Levels of APPs can be significant auxiliary indicators of viral infections, thus the monitoring of their levels in blood serum or in milk can help in early diagnosis. Given the great variability of physiological resting values of APP in farm animals, their application as auxiliary diagnostic tools in viral diseases should only be considered after species- or even breed-specific reference values have been established. This would not only clarify the present situation of APP as an auxiliary diagnostic method for ruminant viral diseases, but also aid in differential diagnostics of other diseases (e.g., bacterial).

In all the viral diseases described in this review, the expression of Hp in blood serum was elevated, and in the most of them, a simultaneous increase in the levels of SAA and Hp was also noted. This finding may suggest that these two proteins are the main APPs in ruminants experiencing viral infections, but since they are elevated in so many different infections, their levels cannot be used to diagnose one infection versus another. Decreased Cp and albumin expression could also help to differentiate BTV infection from the other diseases. For diagnosing an SRLV infection, the analysis of SAA levels in blood serum and milk could be helpful. Despite these possibilities, we conclude that with the current knowledge of the field, APPs should only be used as an auxiliary diagnostic tool in viral diseases.

## Figures and Tables

**Table 1:** Changes in acute phase protein (APP) levels in viral diseases of dairy ruminants.

Virus	Disease	Family of Virus	Species	Positive APP	Negative APP	Ref.
SRLV	Caprine arthritis encephalitis	*Retroviridae*	goat	Hp, SAA	NT	[34]
BDV	Border disease	*Flaviviridae*	goat	Hp, SAA	NT	[35]
PPRV	Peste des petits ruminants	*Paramyxoviridae*	sheep	Hp, SAA	NT	[36]
BTV	Blue tongue disease	*Reoviridae*	sheep	Hp, Cp	albumins	[37,38]
BRSV	Bovine respiratory disease	*Paramyxoviridae*	cattle	Hp, SAA	NT	[39]
BVDV	Bovine viral diarrhea	*Flaviviridae*	cattle	Hp, SAA, Fb	NT	[40,41,42,43]
FMDV	Foot and mouth disease	*Picornaviridae*	cattle	Hp, SAA, Fb, Cp	albumins	[44,45,46]

Hp—haptoglobin, SAA—serum amyloid A, Fb—fibrinogen, Cp—ceruloplasmin, SRLV—small ruminant lentivirus, BDV—border disease virus, PPRV—peste des petits ruminants virus, BTV—bluetongue virus, BRSV—bovine respiratory syncytial virus, BVDV—bovine viral diarrhea virus, FMDV—foot and mouth disease virus, NT—no tested.

**Table 2:** The concentrations of acute phase proteins in healthy and virus-infected dairy ruminants.

Species	Virus	Disease	Health State	SAA	Hp	Cp	Fb	Albumins	References
goat	SRLV	Caprine arthritis encephalitis	Healthy	0.28–27.40 mg/L	0.21–4.89 g/L	NT	NT	NT	[34]
			Infected	0.31–28.70 mg/L	0.22–4.65 g/L	NT	NT	NT	
	BDV	Border disease	Healthy	6.06 µg/mL	0.056 mg/mL	NT	NT	NT	[35]
			Infected	42.13–68.86 µg/mL	0.424–0.866 mg/mL	NT	NT	NT	
sheep	PPRV	Peste des petits ruminants	Healthy	12.80 ^a^	1.54 ^a^	NT	NT	NT	[36]
			Infected	32.3 ^a^	3.13 ^a^	NT	NT	NT	
	BTV	Blue tongue disease	Healthy	ND	0.04 g/L	41.53 mg/dL	NT	ND	[37,38]
			Infected	ND	1.58 g/L	25.59 mg/dL	NT	ND	
cattle	BRSV	Bovine respiratory disease	Healthy	<17 µg/mL	<18 µg/mL	NT	NT	NT	[39]
			Infected	60–80 µg/mL	8–10 mg/mL	NT	NT	NT	
	BVDV	Bovine viral diarrhea	Healthy	25.6 mg/L	0.13 g/L	NT	6.45 g/L	NT	[43]
			Infected	77.7–375 mg/L	0.89–1.87 g/L	NT	6.5–10 g/L	NT	
	FMDV	Foot and mouth disease	Healthy	4.50–4.86 µg/mL	0.084–0.09 g/L	0.06–0.08 g/L	0.06 g/L	3.43 g/dL	[44,46]
			Infected	28.8–45.44 µg/mL	0.308–0.41 g/L	0.10–0.16 g/L	4.64 g/L	2.49–3.39 g/dL	

Hp—haptoglobin, SAA—serum amyloid A, Fb—fibrinogen, Cp—ceruloplasmin, SRLV—small ruminant lentivirus, BDV—border disease virus, PPRV—peste des petits ruminants virus, BTV—bluetongue virus, BRSV—bovine respiratory syncytial virus, BVDV—bovine viral diarrhea virus, FMDV—foot and mouth disease virus, a—no information on units, ND—no differences, NT—no tested.
